# Tracing Fasting Glucose Fluxes with Unstressed Catheter Approach in Streptozotocin Induced Diabetic Rats

**DOI:** 10.1155/2014/743798

**Published:** 2014-03-17

**Authors:** Shichun Du, Hui Wu, Xiao Xu, Ying Meng, Fangzhen Xia, Hualing Zhai, Yingli Lu

**Affiliations:** ^1^Institute and Department of Endocrinology and Metabolism, Shanghai Ninth People's Hospital Affiliated to Shanghai Jiao Tong University School of Medicine, Shanghai 200011, China; ^2^Department of Endocrinology, Zhejiang Province People's Hospital, Hangzhou 310014, China

## Abstract

*Objective*. Blood glucose concentrations of type 1 diabetic rats are vulnerable, especially to stress and trauma. The present study aimed to investigate the fasting endogenous glucose production and skeletal muscle glucose uptake of Streptozotocin induced type 1 diabetic rats using an unstressed vein and artery implantation of catheters at the tails of the rats as a platform. * Research Design and Methods*. Streptozotocin (65 mg**·**kg^−1^) was administered to induce type 1 diabetic state. The unstressed approach of catheters of vein and artery at the tails of the rats was established before the isotope tracer injection. Dynamic measurement of fasting endogenous glucose production was assessed by continuously infusing stable isotope [6, 6-^2^H_2_] glucose, while skeletal muscle glucose uptake by bolus injecting radioactively labeled [1-^14^C]-2-deoxy-glucose. * Results*. Streptozotocin induced type 1 diabetic rats displayed polydipsia, polyphagia, and polyuria along with overt hyperglycemia and hypoinsulinemia. They also had enhanced fasting endogenous glucose production and reduced glucose uptake in skeletal muscle compared to nondiabetic rats. * Conclusions*. The dual catheters implantation at the tails of the rats together with isotope tracers injection is a save time, unstressed, and feasible approach to explore the glucose metabolism in animal models in vivo.

## 1. Introduction

Type 1 diabetes mellitus is a disease of insulin insufficiency that results from the autoimmune destruction of pancreatic *β*-cell in vivo [1]. It is a lifelong disorder depending on exogenous insulin replacement and prone to acquire serious acute and chronic complications [2]. So, it is urgent to clarify the in vivo glucose fluxes to help establish data for treatment of type 1 diabetic mellitus. Streptozotocin (STZ) induced diabetic Sprague-Dawley rats developed the classic trio of symptoms of polydipsia, polyphagia, and polyuria along with overt hyperglycemia and had been widely used for the model of type 1 diabetes in many previous investigations [3–6].

The blood glucose of type 1 diabetic rats fluctuates substantially, especially responding to stress and trauma; furthermore, the type 1 diabetic rats are easy to get infection which inevitably aggravates the hyperglycemia. It is a simple procedure and can be carried out in any laboratory to draw blood sample from the cut tail tip; however, this approach certainly causes huge stress to the rat during the process of the trial and the sample volume is limited [7]. Some previous investigators obtained blood samples from implantation of catheters at the carotid artery of the animals, which entailed general anesthesia and invasive surgery [7–9]. Such a relative complicated process inevitably induced severe glucose fluctuation and enhanced infectious chance and even more mortality in vulnerable STZ induced type 1 diabetic rats. In this study, we chose to use an easy, time saving, and unstressed approach of vein and artery implantation of catheters at the tails of the rats as a platform together with isotope tracer injection to trace the fasting glucose fluxes in STZ induced type 1 diabetic rats in vivo.

## 2. Materials and Methods

### 2.1. Animals and Experimental Groups

20 adult male Sprague-Dawley rats were housed at an ambient temperature of 22 ± 2°C and maintained under a normal 12 hr light-dark cycle with free access to food (52% carbohydrate, 22.1% proteins, 9.2% water, 5.28% fat, 4.12% cellulose, and 4.22% mineral salts) and water. Animals were randomly assigned to two groups (T1D and control group, 10 rats each group). At the 10 weeks of age, animal models of type 1 diabetes were prepared by intraperitoneal injection with STZ (65 mg·kg^−1^) body weight, Sigma, St. Louis, USA) dissolved in the 50 mmol/L sodium citrate buffer (pH 4.5) to irreversibly destroy pancreatic *β* cells after fasting for 8 h. Control rats were injected 50 mmol/L sodium citrate buffer (pH 4.5). Eight rats in T1D group developed diabetes with nonfasting blood sugar >16.7 mmol/L one week later. Two of the ten STZ induced rats died, one died from hypoglycemia the day after the STZ injection and another due to serious intraperitoneal infection. The plasma glucose concentrations were monitored every week by the glucose oxidase method (Yellow Springs Instruments Incorporated, Yellow Springs, OH, USA). The feed and water intake together with body weight of the animals were recorded. At 6 weeks after treatment with STZ or sodium citrate buffer, blood samples were drawn at nonfasting state, centrifuged at 4°C, separated, and stored at −20°C until plasma insulin concentrations were measured. The plasma insulin concentrations were determined using an enzyme-linked immunosorbent assay kit (Diagnostics Systems Laboratories, TX, USA). All the animal procedures were performed in accordance with the ethical principles in animal research adopted by the Department of Laboratory Animal Science, Jiaotong University School of Medicine, Shanghai, China.

### 2.2. Establishment of Unstressed Vein and Artery Implantation of Catheters at the Tails of the Rats

At 6 weeks after treatment with STZ or sodium citrate buffer, rats were fasted for 8 hr. After successfully subcutaneously anaesthetized (1% lidocaine) at the tail root (not obviously struggling when the tip of the tail was nipped with forceps), the rat was placed with back up. The operator made the lateral vein more obvious by applying warm water and alcohol wiper at the lower 1/3 part of the tails and then carefully inserted the catheter with stiletto (24 G × 0.75 IN, BD Insyte, USA) in the vein at an angle about 30°. If successfully placed, the catheter could be seen with vein blood flowing out gradually and continuously after the operator withdrew the stiletto. The vein catheter then was closed up with a heparin tap and fixed on the tail with sticky tape.

After the implantation of the vein catheter, rat was fixed with abdomen up. The operator made a longitudinal incision at the upper 1/3 of the tail, carefully isolated the subcutaneous tissue and made a small hole on the sheet of the caudal artery with eye-surgical scissors to expose approximately 5 mm of the artery. The operator then placed a silk suture underneath the artery for later use, nipped the proximal part of the artery with forceps, and pulled a little backward to keep straight and maintain some tension of the artery. Another catheter with stiletto (22 G × 1.0 IN, BD Insyte, USA) was inserted into the caudal artery at an angle about 15° with a quick and careful motion. The catheter could be seen instantly congested with artery blood after the stiletto was retreated a little bit but still in the catheter if the catheter was successfully placed in the artery. The operator then inserted the catheter further (about 1 cm) with a parallel angle in the artery and withdrew the stiletto totally if the catheter was satisfactorily placed in the artery. The catheter was closed up with a heparin tap and anchored with artery using the silk suture underneath. The subcutaneous tissue as well as the skin then was sewn up with 3-0 silk suture. After the implantation of the V-A catheters, the rat was transferred into a self-made metabolic cage where it could eat water, rest, or move mildly with the tail fixed by a self-made adjustable copper clamp to match different diameters of the tails. Saline (10 *μ*L/min) was consistently injected through vein catheter by a Harvard pump (Harvard Apparatus, Holliston, MA, USA) to maintain the patency of the vein injection.

### 2.3. Measurement of Fasting Hepatic Glucose Production and Skeletal Muscle Glucose Uptake by Isotope Tracer

Before initiation of the tracers, rats needed to relax in the metabolic cage for about 15–30 min until the blood glucose concentrations were constantly stable. In order to determine the rate of fasting endogenous glucose production, [6, 6-^2^H_2_] glucose (Cambridge Isotope Laboratories, 99% of purity) was consistently infused (2 *μ*mol·kg^−1^·min^−1^) from time 0 by another Harvard pump through the vein catheter for 120 min. [1-^14^C]-2-deoxy-glucose (PerkinElmer Life Sciences) was administered as a bolus (1 *μ*Ci) with isotonic saline before 30 min of the end of the study (time 90) to measure the fasting skeletal muscle glucose uptake. Blood samples (each 0.5 mL) were collected at time 0 (before [6, 6-^2^H_2_] glucose injection), time 100, 110, and 3 min intervals before the end of the study (time 114, time 117, and time 120) from the tail arterial catheter. At the end of the study (time 120), the rats were anesthetized by intravenously injected with 3% pentobarbital sodium (50 mg·kg^−1^) and gastrocnemius muscle was obtained and immediately frozen at liquid nitrogen for the measurement of ^14^C specific radioactivity in a liquid scintillation counter (LS 6000TA; Beckman Instruments).

### 2.4. Measurement of Stable Isotope Enrichment Using Gas Chromatography/Mass Spectrometry

Plasma samples drawn during the tracing trial were processed by methoxyamin-HCL, BSTFA, TMCS, and pyridine. Samples were run on gas chromatography/mass spectrometry (GC-MS, Agilent 5975C, Agilent Technologies). The MS was operated in the selected ion monitoring (SIM) mode monitoring fragments at mass to charge ratios (m/z) 319.20, 321.20 for unlabeled and [6, 6-^2^H_2_] glucose. GC operating condition was 70°C for 4 min, 70°C to 240°C at 10°C/min, 240–300°C at 20°C/min, remained at 300°C for 11 min, and constant flow conditions [11].

### 2.5. Calculation of Fasting Endogenous Glucose Production (EGP) and Skeletal Muscle Glucose Uptake

Fasting endogenous glucose production (*μ*mol·kg^−1^·min^−1^) was calculated by dividing the [6, 6-^2^H_2_] glucose infusion rate (*μ*mol·kg^−1^·min^−1^) by the steady-state concentration ratio of [6, 6-^2^H_2_] glucose (99% of tracer) divided by tracee plus tracer glucose. The skeletal muscle glucose uptake (*μ*mol·g^−1^·min^−1^) was determined by ^14^C specific radioactivity per gram of the muscle sample (dpm·g^−1^) divided by the AUC from 90 to 120 min of plasma ^14^C specific activity (dpm·min·*μ*mol^−1^).

### 2.6. Statistical Analysis

The results are expressed as mean ± SD. The unpaired Student's* t*-test was used for analyzing the data between two groups. A value of *P* < 0.05 was considered statistically significant.

## 3. Result

### 3.1. Features of STZ Induced Type 1 Diabetic Rats

**Figure 1:**
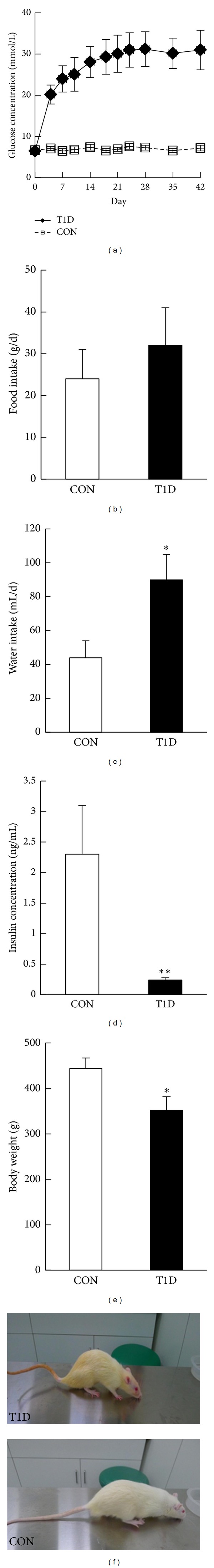
(a) Plasma glucose concentrations were observed for the 6 weeks after STZ or sodium citrate buffer treatment. (b and c) Intake of food and water per day in type 1 diabetic (T1D group) and nondiabetic rats (control group) after 6 weeks of treatment with STZ. (d) Nonfasting plasma insulin concentrations in type 1 diabetic and nondiabetic rats after 6 weeks of treatment with STZ. (e and f) T1D rats showed significantly reduced body weight along with dismal hair, irritability, and weakness after 6 weeks of treatment with STZ. **P < 0.01, *P < 0.05 between the two groups.

STZ treatment rapidly produced the characteristic signs of type 1 diabetes such as sustained increased nonfasting plasma glucose concentration (30.1 ± 4 versus 7.2 ± 0.5 mmol/L, Figure 1(a)), intake of both food (32 ± 9 versus 24 ± 7 g/d, *P* > 0.05, Figure 1(b)) and water (90 ± 15 versus 44 ± 10 mL/d, *P* < 0.05, Figure 1(c)) as well as significant decreased nonfasting plasma insulin concentrations (0.24 ± 0.04 versus 2.3 ± 0.3 ng/mL, *P* < 0.01, Figure 1(d)) as compared to the control rats. At the end of 6 weeks of treatment with STZ, the T1D rats showed significantly reduced body weight (352 ± 30 versus 444 ± 23 g, *P* < 0.05, Figure 1(e)) along with dismal hair, irritability, and weakness compared to the control group (Figure 1(f)).

### 3.2. Unstressed Vein and Artery Implantation of Catheters at the Tails of the Rats

**Figure 2:**
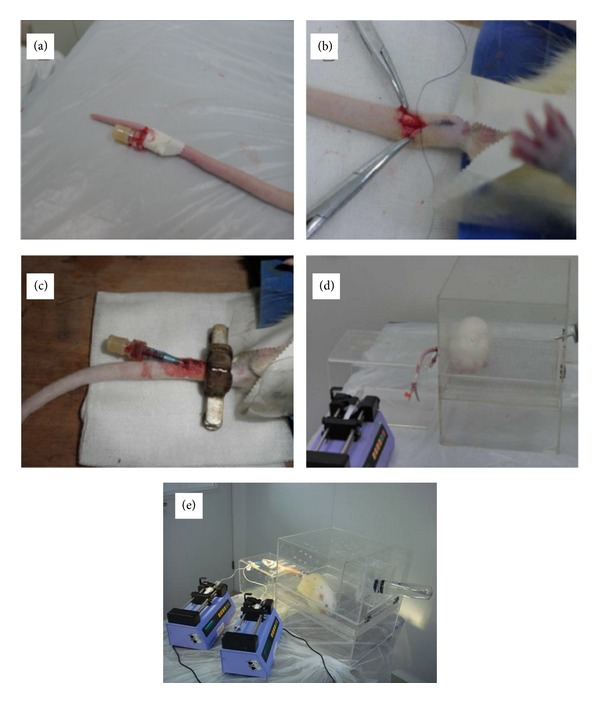
Establishment of platform of unstressed vein and artery implantation of catheters at the tails of the rats. (a) A catheter was implanted in the vein of the rat tail. (b and c) Another catheter was implanted into the artery of the rat tail with an adjustable copper clamp fixed at the tail root. (d) The rat was transferred into a self-made metabolic rage to rest before tracing study. (e) The unstressed tracing model with vein continuously injected tracers and artery catheters for drawl of blood samples (published by Zhai et al. [16]).

Figures 2(a)–2(e) show the procedure of the unstressed implantation of dual catheters at the tail of the rat. The process of implantation of dual catheters was successfully finished about 15–30 min by a skilled investigator. During the process of tracing, the tracer room maintained quiet with ambient temperature of 22 ± 2°C. Rats were relaxed, resting, sleeping, or drinking water ad lib. The stress during the tracer trial was extremely minimized (Figure 2(e)).

### 3.3. Fasting Endogenous Glucose Production and Skeletal Muscle Glucose Uptake of the Type 1 Diabetic Rats

**Figure 3:**
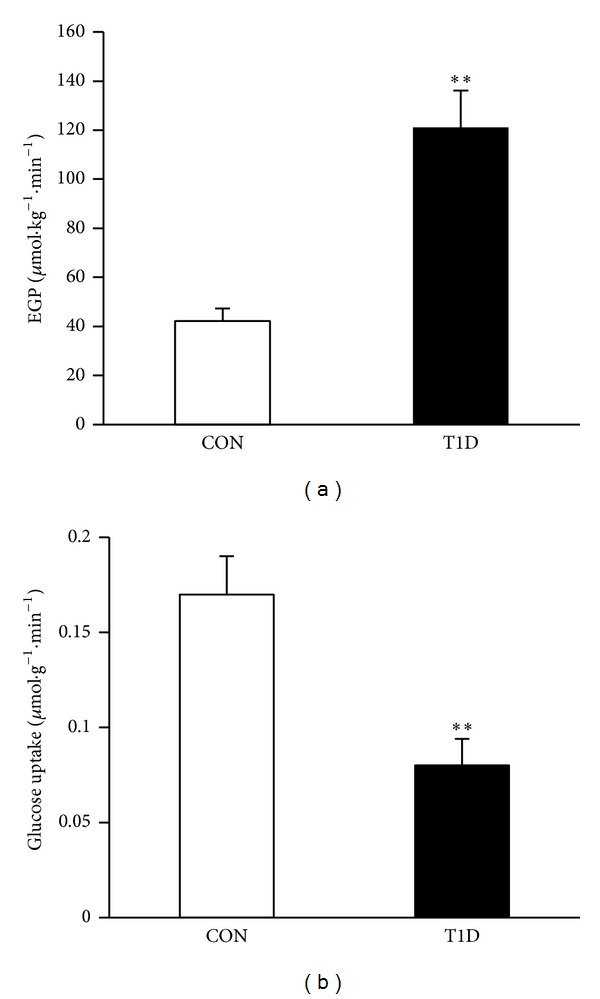
Measurement of fasting endogenous glucose production (EGP, (a)) and skeletal muscle glucose uptake (b) in type 1 diabetic and nondiabetic rats. **P < 0.01 between the two groups.

Fasting EGP value at time 114 (120 ± 19 and 46 ± 11 *μ*mol·kg^−1^·min^−1^ in T1D and control rats), time 117 (126 ± 15 and 40 ± 8 *μ*mol·kg^−1^·min^−1^ in T1D and control rats), and time 120 (118 ±11 and 51 ± 12 *μ*mol·kg^−1^·min^−1^ in T1D and control rats) was stable which suggested that the steady state (plasma glucose appearance rate equals disappearance rate) had arrived. The fasting endogenous glucose production in T1D group was significantly increased compared to those in the control group (121 ± 15 versus 45 ± 10 *μ*mol·kg^−1^·min^−1^, *P* < 0.01, Figure 3(a)) after about 8 h of fasting. The fasting glucose uptake in gastrocnemius muscle was significantly reduced in T1D group compared to the control group (0.08 ± 0.01 versus 0.17 ± 0.02 *μ*mol·g^−1^·min^−1^, *P* < 0.01, Figure 3(b)).

## 4. Discussion

Type 1 diabetic patients are prone to develop severe acute and chronic complications which inevitably cause disability and mortality [12, 13]. Thus, it is important to trace the glucose fluxes of T1DM to elucidate the pathogenic mechanism using animal models. Streptozotocin (STZ) is an antibiotic that can cause pancreatic **β**-cell destruction and widely used experimentally as an agent capable of inducing type 1 diabetes mellitus [14, 15].

In the previous investigations, in order to dynamically trace the glucose fluxes in the rodent animals in vivo, catheters for infusion of substrates and/or withdrawal of blood samples were inserted into the right jugular vein and left carotid artery, with the catheters connected with medical tubing suspended overhead [8]. The rats had to be administered general anesthesia prior to implantation of catheters and a major surgery afterwards. It is reported that plasma glucose concentration may rise by 10–20 mg/dl or more in nondiabetic rats in comparison to nonanesthetic ones because of the plasma catecholamine and other counter-insulin hormone released during and after the surgery [7, 8]. After surgery, rats need to rest for at least 4–6 days to start metabolic research. During that time, some will suffer recatheter because of obstruction of the original ones. Vulnerable T1DM rats will suffer a more critical time of weight loss, blood glucose fluctuation, and some even die from infections or acute diabetic complications.

In order to minimize the undesirable influence of surgical trauma and alleviate the surgery complication of the catheter, we have utilized an unstressed experimental model together with isotope tracer technique to describe the glucose fluxes in the conscious rats. Our approach and device were more easy, nonstress, and decreased severe trauma and blood glucose fluctuation to a large extent (Table 1). Guo and Zhou [10] have presented similar technique in normal rats and argued this catheterization approach greatly reduced the labor and saved time expended in implanting catheters and raising animals during the recovery period. We found through this and previous studies using the dual tail catheters conducted in our laboratory [16, 17] that the animals usually slept or rested calmly and drank ad libitum during the whole experiment (Figure 2(e)). Instead of chronic investigations, we designed our unstressed tail V-A catheters approach for acute, convenient, and practical implication. In order to maintain the patency of the catheters, saline were constantly and slowly infused through the vein catheter during the whole study and heparinized saline (20 U/mL) were injected into the artery catheter cavity right after every blood draw. So we did not have the question of catheters blockage during the study. After the study, the catheters were removed from the animals. Up to now, we have not investigated the chronic side-effect of the implanted catheters, such as blockage or infection rate after several days. However, we believe our rat catheter model can be used for chronic purpose if anticoagulant drugs and additional shielding appropriately implicated. In our later studies, we shall extend the implication of the unstressed tail V-A catheters of rats together with isotope tracer for further prolonged or complicated studies.

Stable isotope [6, 6-^2^H_2_] glucose injected with a minute amount to trace the natural fasting glucose fluxes without influence plasma glucose concentration is a classical approach to research glucose mechanism in animals and humans in vivo [16, 17, 21, 22]. In type 1 diabetic animals as well as humans, exhausted insulin production result in the nonsuppressible secretion of glucagon which lead to increased endogenous glucose production eventually. In our trace study, we found significantly elevated fasting endogenous glucose production in type 1 diabetic rats compared to nondiabetic rats, which is a direct proof of elevated fasting plasma glucose concentration [23–25].

Glucose needs to be phosphorylated by hexokinase inside the muscle cell before it enters the steps of glycogen synthesis and/or breakdown. Phosphorylated [1-^14^C]-2-deoxy-glucose is not a substrate for phosphohexose isomerase, so it accumulates and is not utilized by the muscle, and it is frequently measured as an index of muscle glucose uptake [26–28]. Skeletal muscle is the most important tissue for glucose disposal after a meal or high plasma glucose level with the effects of insulin. However, the lack of insulin in type 1 diabetic rats impairs glucose uptake of the skeletal muscle. Using this tracer method to assess muscle glucose metabolism, we got the result that muscle glucose uptake was significantly decreased in type 1 diabetic rats than nondiabetic ones. New drugs which have potential beneficial effect on the muscle glucose uptake of type 1 diabetic rats may be tested further implication using this approach.

Our data of EGP and glucose uptake in diabetic and normal rats were in accordance with Youn et al. [25] who conducted hyperinsulinemic glucose clamps in rats without (control) and with STZ-induced diabetes (the data at 2 weeks after STZ) using [3-^3^H] glucose tracer infusion. The EGP was 10 ± 7 *μ*mol·kg^−1^·min^−1^ in normal rats and 108 ± 9 *μ*mol·kg^−1^·min^−1^ in diabetic rats during the hyperinsulin clamps, which suggested that insulin greatly suppressed the EGP in normal rats, while the effects were limited in the type 1 diabetes rats. Glucose uptake was about 50% less in T1D than control (0.125 ± 0.005 versus 0.246 ± 0.015 *μ*mol·g^−1^·min^−1^), which were comparable to our results.

The unstressed vein and artery implantation of catheters at the tail of the rats together with isotope tracer injection of the study provide a dynamic description of glucose kinetics in the type 1 diabetic rat models in fasting state. The useful platform certainly will have important implications with further modification for conducting insulin clamp trials as well as for the drug, glucose, and lipid metabolism research in a variety of animal models in vivo.

## Figures and Tables

**Table 1:** Comparison of conventional catheterization and unstressed tail vein-artery catheterization in normal SD rats.

	Conventional catheterization	Unstressed tail catheterization
Trauma	Profound	Minimal
Anaesthetic	General	Local
Rest after surgery	4–6 d	15–30 min
Weight loss	Obvious	Not obvious
Blood glucose fluctuation	Elevated 10–20 mg/dL	Not obvious
10 d blockade rate	15–25%	NA
Infection rate	Up to 30%	NA

Conventional catheterization: jugular vein and carotid artery catheterization. The data of conventional catheterization were extracted from references [7, 8, 18–20]. NA: chronic data not available.
